# Insulin Mimetic Peroxo Complexes of Vanadium Containing Uracil or Cytosine as Ligand

**DOI:** 10.1155/MBD.2000.157

**Published:** 2000

**Authors:** Asit R. Sarkar, Shipra Mandal

**Affiliations:** Department of Chemistry University of kalyani Kalyani W.B. 741235 India

## Abstract

Mixed ligand oxo peroxo complexes of vanadium (V), M[VO(O_2_)L_2_].nH_2_O where M = K or NH_4_, HL = uracil or cytosine and n = 1 or 2, have been isolated from aqueous methanolic medium. The complexes were characterised by elemental analysis, conductance, TGA, UV-Visible, IR and NMR spectral studies. Both the peroxide and the other ligands acts as chelates coordinating through their oxygen at C (2) and nitrogen at N (3) and the presence of monomeric oxoperoxovanadium (V) species, have been established by IR, ^1^H and ^51^V NMR studies. The complexes appeared to possess pentagonal bipyramidal geometry. The terminal oxo ligand and the oxygen of the uracil or cytosine at C (2) occupy the axial position while the peroxide and the other donor ligands are in the equatorial position. The UV spectral studies confirm the presence of Vanadium in its +5 oxidation state. The administration of the potassium salts of the complexes reduces the blood glucose level in Swiss Albino mice compared to that of KVO_3_. The complexes also readily oxidise cysteine to Cystine in aqueous solution. The possible mechanism for the insulin-mimic activity of the complexes is discussed.


### INSULIN MIMETIC PEROXO COMPLEXES OF VANADIUM CONTAINING URACIL OR CYTOSINE AS LIGAND

Asit R. Sarkar* and Shipra Mandal

Department of Chemistry, University of Kalyani, Kalyani- 741235, W.B. India

### ABSTRACT

Mixed ligand oxo peroxo complexes of vanadium (V), $M[VO(O_2)L_2].nH_2O$ where M = K or $NH_4$, HL = uracil or cytosine and n = 1or 2, have been isolated from aqueous methanolic medium. The complexes were characterised by elemental analysis, conductance, TGA, UV-Visible, IR and NMR spectral studies. Both the peroxide and the other ligands acts as chelates coordinating through their oxygen at C (2) and nitrogen at N (3) and the presence of monomeric oxoperoxovanadium (V) species, have been established by IR, $^1H$ and $^{51}V$ NMR studies. The complexes appeared to possess pentagonal bipyramidal geometry. The terminal oxo ligand and the oxygen of the uracil or cytosine at C (2) occupy the axial position while the peroxide and the other donor ligands are in the equatorial position. The UV spectral studies confirm the presence of Vanadium in its +5 oxidation state. The administration of the potassium salts of the complexes reduces the blood glucose level in Swiss Albino mice compared to that of $KVO_3$. The complexes also readily oxidise cysteine to cystine in aqueous solution. The possible mechanism for the insulin- mimic activity of the complexes is discussed.

### INTRODUCTION

The discovery of the insulin mimetic properties of vanadium (V) compounds has increased the interest in the studies of the structure and reactivity of vanadium (V) complexes[1]. The enhanced insulin mimetic effect of the peroxo vanadium complexes has further increased the interest in the coordination chemistry of vanadium[2-4]. The vanadium peroxo complexes are probably formed as intermediates in oxidation reaction catalysed by vanadium bromo peroxidase, an enzyme from marine algae[5]. Vanadium peroxo complexes have also been used in synthetic organic chemistry since these compounds have potent and very desirable properties as catalysts[6,7]. As a result, the studies on these vanadium peroxo complexes have appeared in several recent reviews[8,9]. The insulin mimic property of vanadium (V) compound was first reported in 1980[10]. Subsequently, it was reported that mixtures of hydrogen peroxide and vanadates act as a more potent insulin mimetic agent in controlling the blood glucose level in rats, than, either vanadates or hydrogen peroxide[1,11]. The mixtures of vanadates and hydrogen peroxide generate several peroxovanadium species[12] in aqueous solution and the mixtures also become unstable losing their insulin mimetic activities with time[2]. Mixed ligand peroxo vanadium complexes have been isolated[2] in solid state with pyridine-2-carboxylic acid or with 1,10-phenanthroline as ligands, which exhibit, improved insulin mimetic activity[13]. Thus mixed ligand peroxo complexes of vanadium, containing N- and O-chelating donor ligands act as potent insulin mimetic agents. A new insulin mimetic peroxo vanadium compound containing imidazole[13] and naturally occuring chelating oxazolinate or thiazolinates has been reported[14] recently. Recent investigations of vanadium compounds as antidiabetic drugs in human have enhanced the interest in both the chemistry and in the biological mechanism of actions of vanadium[15, 16]. The nucleic acid bases such as uracil and cytosine possess the N- and O-donor ligands. But there is no report on the mixed ligand peroxo complexes of vanadium containing these ligands though they themselves are of biological interest[17,18]. Both uracil and cytosine can undergo tautomeric changes as shown in Fig. 1.

Hence, in continuation to our work on the interactions of the nucleic acid bases with metal ions[19,20], we have undertaken the present investigation and report here the isolation and characterisation of four novel mixed ligand peroxo vanadium complexes containing uracil or cytosine as ligands. All the prepared complexes may be represented as $M[VO(O_2)L_2].nH_2O$, where M = K or $NH_4$, HL = uracil or cytosine and n = 1 or 2.

### MATERIALS AND METHODS

Ammonium vanadate, uracil, cytosine were from Aldrich Chemical Company, USA. Vanadium pentoxide was obtained from Sigma, USA. The other chemicals used were of analytical grade. The uracil or cytosine is resistant to oxidation by $H_2O_2$ under the experimental conditions.

Preparation of the Complexes

**$K[VO(O_2)(ura)_2].H_2O$, (1):** 0.91g (5.0 mmol) of $V_2O_5$ dissolved in 0.65 g (11.6 mmol) of KOH in 10 ml of water. A clear pale green solution was obtained. The solution was cooled. To this solution previously cooled (5° C) 30 % $H_2O_2$ (1.5 ml, 13.2 mmol) was added and the resulting solution became yellow. The mixture was cooled to 5° C. 2.5 g (22.3 mmol) of uracil (Hura) dissolved in minimum volume of aqueous methanol (50 %) was added to the above cooled mixture.

The color of the resulting solution turned orange yellow. The pH of the solution was brought around 7.5 by adding dropwise 0.1 (M) NaOH solution. The resulting solution was stirred for 1 hour and cooled to 5° C. A yellow precipitate was obtained that was recrystallised from 3 % $H_2O_2$ solution. (Yield: 1.6 g, 42.1 %). Anal Found: V, 13.87; C, 25.12; H, 2.52; N, 14.94 %. Calculated for $C_8H_8KN_4O_8V$ (F.W 378.1), V, 13.48; C, 25.39; H, 2.11; N, 14.81 %.

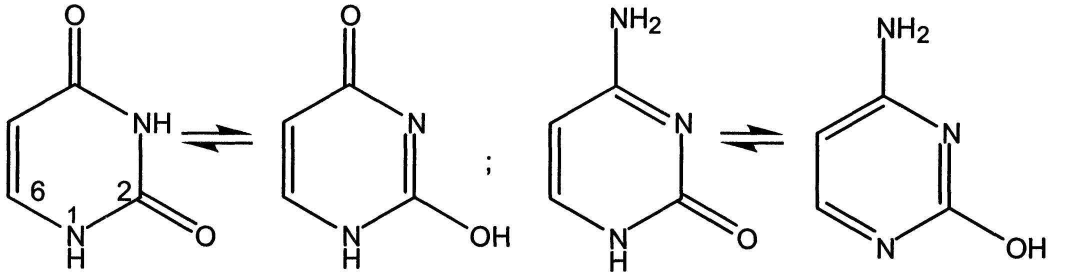

Fig. 1: Tautomeric forms of uracil (a) and cytosine (b), and atoms numbering

**$K[VO(O_2)(cyt)_2].H_2O$ (2):** The yellow compound was obtained in the same way as adopted for the above compound **(1)** except that 2.5 g (22.5 mmol) of cytosine (Hcyt) was used instead of uracil. The yellow product was recrystallised from 3 % $H_2O_2$ solution. (Yield: 1.5 g, 39.9 %). Anal. Found: V, 13.62; C, 25.26; H, 2.73; N, 22.59 %. Calculated for $C_8H_{10}KN_6O_6V$ (FW = 376.1), V, 13.56; C, 25.53; H, 2.66; N, 22.33 %.

**$NH_4[VO(O_2)(ura)_2].2H_2O$, (3):** 0.59 g (5.0 mmol) of $NH_4VO_3$ was dissolved in 15 ml 6% $H_2O_2$ (26.4 mmol). The resulting solution, coloured yellow, was cooled to 5° C. To this 2.5 g (22.3 mmol) of uracil (Hura) dissolved in methanol was added. The colour of the resulting solution became orange yellow. The pH of the solution was brought around 7.5 by adding 0.1 M NaOH dropwise. The colour of the solution turns bright yellow. The solution was well stirred for 1hour and was kept at 5° C. Yellow precipitate was obtained which was recrystallysed from 3% $H_2O_2$ solution.(Yield: 0.6 g, 32%). Anal. Found: V, 13.68; C, 25.86; H, 3.97; N, 18.93. Calculated for $C_8H_{14}N_5O_9V$ (F.W 374.94), V, 13.59; C, 25.60; H, 3.73; N,18.67 %.

**NH4[VO(O2)(cyt)$_2$].2H$_2$O (4):** The compound was obtained in the same way as adopted for the above compound **(3)** except that 2.5 g (22.5 mmol) of cytosine (Hcyt) was used instead of uracil, all other conditions remaining the same. The yellow compound was recrystallised from 3% $H_2O_2$ solution. (Yield: 0.5 g, 26.3%). Anal. Found: V, 13.74; C, 27.02; H, 4.40; N, 26.13 %. Calculated for $C_8H_{16}N_7O_7V$ (FW, 373), V, 13.67; C, 26.74; H, 4.29; N, 26.27 %.

Analysis

Carbon, hydrogen and nitrogen microanalyses were obtained from the Indian Association for the Cultivation of Science, Calcutta. Vanadium was estimated [21] by titration with a $Fe^{2+}$ solution in the presence of sulphonated diphenylamine as indicator, after decomposing the sample with sodium peroxide. The peroxo content was determined by titration of a freshly prepared solution of the compounds with potassium permanganate in 2 (N) sulphuric acid.

Physical Measurements

$^1H$ NMR spectra of the potassium salts of the complexes were obtained in $D_2O$ at 19° C with a JEOL FT-100 NMR spectrometer using HOD at 4.63 ppm as reference. The $^{51}V$ NMR spectra of the complexes were recorded in $D_2O$ with a Varian XL-300 NMR spectrometer using $VOCl_3$ as external standard. The UV visible spectra were recorded either in a solid mull or in aqueous solution using a DMR 21 Karl Zeiss spectrophotometer and the ir spectra were recorded in KBr pellets by means of a 1330 Perkin-Elmer spectrophotometer. The conductivity measurements were carried out with a PR 9500 Philips conductivity bridge. A thermogravimetric analysis was carried out with a Derivatograph (system: F.Paulik, J. Paulik, L. Erday. MOM, Budapest). About 100 mg of the finely powdered substances was heated at a rate of 2° C per minute.

Estimation of blood glucose level

Blood glucose was estimated in Swiss Albino mice, weighing around 30 g each spectrophotometrically with ortho-toluidene as the reagent[22]. A calibration curve was recorded with standard glucose solution of varying concentration from 25 to 200 $\mu g\ L^{-1}$ containing 3% trichloroacetic acid and ortho-toluidene as the coloring agent. Blood glucose level was measured from the calibration curve after treating the mice with vanadate solution, hydrogen peroxide and the isolated potassium salts of the complexes with varying concentrations. The plasma blood glucose contents of the treated mice and the controlled untreated mice were calculated. The compounds in aqueous solution were administered by a single intravenous tail vein injection of varying concentrations ranging from 0.01 mmol to 0.06 mmol / kg wt in a volume of 1.5 $\mu l\ g^{-1}$ body weight. Analysis of plasma glucose levels of blood was done by nicking the tip of the tail of the mouse and collecting 5 µl blood into the injection syringe. The final volume was adjusted to 7.0 ml by adding 3% trichloroacetic acid solution. The solution was boiled for 10 minutes and then cooled to room temperature for recording the absorbance at 630 nm.

Oxidation of cysteine to cystine

Stock solutions of L-cysteine $1x10^{-1}$ M, compounds solution $5x10^{-2}$ M, potassium vanadate $5x10^{-2}$ M and $H_2O_2$ $1x10^{-1}$ M were prerared in phosphate buffer solution having pH 7.4. Solution of cysteine and the compounds in the molar ratio 1:1, 2:1, and 4:1 were prepared by adding 10 ml of cysteine stock solution to the appropriate amount of oxidant, followed by dilution with buffer to 30 ml total volume. A control sample 10 ml of cysteine solution and 20 ml of buffer was prepared for each experiment. All the reactions were carried out in nitrogen atmosphere and well stirred for 3 hours except those with $KVO_3$ that was allowed to react for 20 hrs. Then the samples were filtered through pre weighed sintered glass filters which were dried under vacuum overnight and reweighed to give the yield of cystine. The cystine produced was identified in each case by comparing with its $^1H$ NMR spectra.

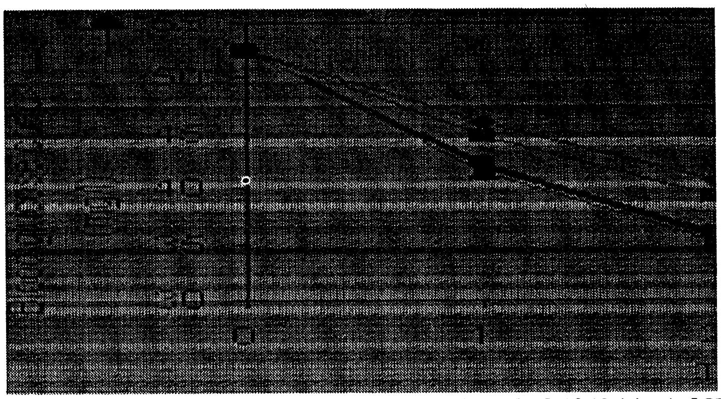

Fig. 2: Plasma blood glucose level on administration of $K[VO(O_2)(ura)_2].H_2O$ at different concentrations and times

### RESULTS

All the compounds are bright yellow in colour, non-hygroscopic and are stable at room temperature. The compounds are soluble in water affording $10^{-3}$ M solution and the molar conductance values lie in the range 108-125 $ohm^{-1}\ cm^2\ mol^{-1}$ indicating the presence of 1:1 electrolytes in aqueous solution. The thermograms of the ammonium salts of the complexes show that they begin to decompose around 75° C and the decomposition takes place gradually without the formation of any stable intermediates. Finally a horizontal is obtained around 450° C with a loss of around 72 % indicating the total conversion of the sample into vanadium pentoxide.

I.R Data

The important ir absorption bands of the complexes are given in table I. All the compounds show a very strong band around 940 $cm^{-1}$ which may be assigned due to O-O stretching of bidentate peroxide group[23]. In addition to that another strong band around 620 $cm^{-1}$ in all the compounds may be assigned due to assymetric metal-peroxide vibrations. The absorption due to V=O vibration around 970 $cm^{-1}$ indicate the high π-bond order of the vanadium-oxygen bond and also the presence of monomeric oxovanadium species[24]. The absorption frequencies of the uracil complexes were compared to that of the free uracil in the region between 1800 $cm^{-1}$ - 1300 $cm^{-1}$ where the CO and NH frequency of uracil is located[25]. The bending vibrations of N(1)-H at 1508 $cm^{-1}$ of uracil in the complex remains almost unchanged both in intensity and position while the vibration due to N(3)-H of the uracil at 1417 $cm^{-1}$ disappears completely in the complexes. The position and intensity of the bands assignable to the 2-keto group at 1716 $cm^{-1}$ of the uracil in the complexes change appreciably with respect to free uracil while there is a little change for the vibrations of 4-keto group. So, in the complexes uracil acts as a chelating ligand binding through its C(2)=O and N(3). The cytosine complexes were also compared with that of the free cytosine as given in the literature[26]. The C-$NH_2$+C-N ring stretching frequency at 1290 $cm^{-1}$ was split and shifted to higher frequency but with reduced intensity. Since this shift is probably caused by the inductive and mesomeric effect of the ring, N(3) site of the cytosine molecule in the complexes is assumed to be more positively charged than the free cytosine molecule, indicating the coordination through N(3) of cytosine[27].

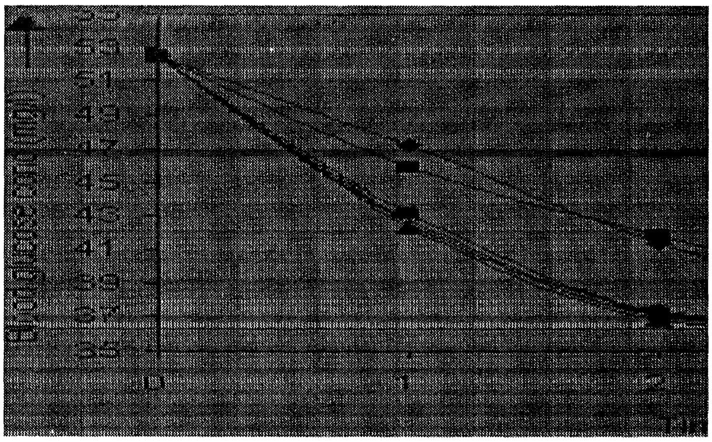

Fig. 3: Plasma blood glucose level on administration of $K[VO(O_2)(cyt)_2].H_2O$ at different concentrations and times

The shoulder at 1660 $cm^{-1}$ and the strong band at 1503 $cm^{-1}$ in cytosine due to the C=N+C=C stretching frequencies were shifted to higher frequencies in the complexes due to the redistribution of π-electrons in the conjugated C=C, C=N and C=O systems of the cytosine ring. This also supports the coordination of the metal through the N(3) of the cytosine[32]. The C=O band of cytosine at 1650 $cm^{-1}$ should shift to higher frequency on coordination because there is no longer electron migration from the N(3) position to C(2). The almost unchanged position of the C=O band both in position and intensity indicates the coordination also through C=O. Hence in these complexes, cytosine also acts as a bidentate ligand coordinating through the nitrogen at N(3) and the oxygen of C=O[28]. The bidentate peroxide ligand in general is bound in the equatorial plane relative to the axial oxoligand in monomeric oxoperoxo vanadium complexes[29]. The oxygen donor of another ligand molecule may serve as the seventh ligand as a long axial bond to possess pentagonal bipyramidal geometry. In mono peroxo complexes the peroxide is symmetrically coordinated.

UV Visible Spectra

The U.V visible spectra of the complexes show two bands. One low intensity band around 332 nm and one high intensity around 225 nm. Both the bands may be assigned due to $\pi^*$ - $d\sigma^*$ electron transitions from the filled $\pi^*$ orbital of the peroxo group to the vacant d orbital of vanadium[30]. The high intensity peak around 225 nm will be due to the $\pi$-$\pi^*$ transitions of the uracil or cytosine ligands and may also contain n-d and $\pi^*$-d transition from the uracil or cytosine ligands to the vacant d - orbital of the vanadium[31]. The absence of d-d transitions in the visible region also establishes the presence of vanadium in its +5 state in the complex.

Table I: Assignment of important IR bands ($cm^{-1}$)

| Hura | Hcyt | (1) | (2) | (3) | (4) | Assignments |
|---|---|---|---|---|---|---|
| 1716 s | 1650 s | 1700 w | 1640 s | 1700 | 1645 s | ν[C(2)=O] |
| | 1660 sh | | 1620 w | | 1625 w | ν[CN+CC] |
| 1675 s | | 1670 m, br | | 1670 m, br | | ν[C(4)=O+C=C] |
| 1508 | | 1510 m | | 1510 m | | δ[N(1)-H] |
| | 1503 s | | 1525 m | | 1525 m | ν[CN+CC] |
| 1417s | | | | | | δ[N(3)-H] |
| | 1290 m | | 1300 vw | | 1300 vw | ν[$CNH_2$+CN] |
| | | 970 vs | 975 vs | 970 vs | 975 vs | ν[V=O] |
| | | 940 s | 935 s | 940 s | 935 s | ν[O-O] |
| | | 620 s | 625 s | 620 s | 625 s | $\nu_{as}[M\text{-}O_2]$ |

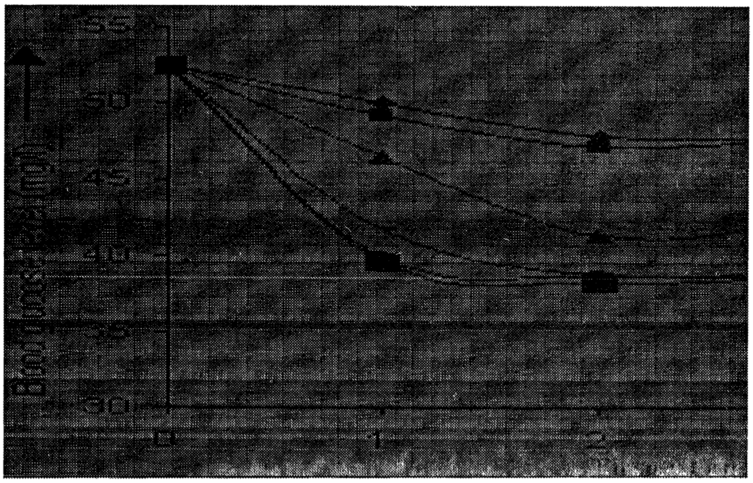

Fig. 4: Plasma blood glucose level on administration of $KVO_3$ at different concentrations and times

NMR Spectra.

Selected $^1H$ NMR signals of uracil, cytosine and the isolated vanadium peroxo complexes containing those ligands are given in Table II. The assignments have been made as reported in the literature[32,33]. The potassium complex containing the monodeprotonated uracil shows the presence of N(1)-H groups (δ=10.20 ppm ). On brining down the pH to around 4 by adding $CF_3COOH$ both H(5) and H(6) signal shows a downfield shift from 5.71 ppm to 5.88 ppm and from 7.38 ppm to 7.44 ppm respectively. The greater downfield shift of the H(5) signal is explained by the protonation of the oxygen at C(4) which is close to H(5) suggesting the presence of free oxygen at C(4) and the co-ordination of the vanadium atom through O at C(2). The downfield shift of the H(6) signal is explained by the protonation at the N(1) site. The NMR spectra itself also indicate the presence of free N(1)-H group. Hence, N(1) and oxygen at C(4) are not involved in bonding.

Table II. $^1H$ and $^{51}V$ NMR signals of the ligand and the complexes (δ, ppm)

| Compound | Solvent | $^1H$ | | | $^{51}V$ |
|---|---|---|---|---|---|
| | | H-C(5) | H-C(6) | H-N(1) | |
| Hura | $D_2O$ | 5.53 (d) | 7.49 (d) | 10.12 (s) | |
| **(1)** | - do - | 5.71 (d) | 7.38 (d) | 10.20 (s) | -582 |
| -do- | $D_2O+CF_3COOH$ | 5.88 (d) | 7.44 (d) | 10.31 (s) | |
| Hcyt | $D_2O$ | 6.81 (d) | 7.78 (d) | 10.08 (s) | |
| **(2)** | - do - | 6.40 (d) | 7.82 (d) | | -588 |
| -do- | $D_2O+CF_3COOH$ | 6.44 (d) | 7.92 (d) | | |

This also supports our observation from the IR spectra that the uracilato anion co-ordinates to vanadium through its O at C(2) and N at N(3). The complex containing the monodeprotonated cytosine anion does not exhibit the signal for N(1)-H compared to that of free cytosine. Further on bringing down the pH around 4 by adding $CF_3COOH$ there is a further downfield shift of the C(5)-H and C(6)-H signals. The downfield shift for C(6)-H (0.10 ppm) is higher compared to that for C(5)-H (0.04 ppm). The greater downfield shift for C(6)-H can be explained due to the protonation of the free N(1) site of the deprotonated cytosine anion in the complex. The $^{51}V$ signal appeared at -582 ppm and at -588 ppm for the above complexes is consistent with the monoperoxo species[34].

BIOLOGICAL TESTS

Administration of our compounds (**1**), (**2**) and potassium vanadate over different concentration and time reduces the plasma glucose level in the mice compared to the controlled mice as presented in Fig. 2 and Fig. 3. The reduction is much more effective in the case of our compounds with respect to potassium vanadate. The optimum dose of our compound is 0˙03 mmol/kg wt for a period of 2 hours which reduces the blood glucose level to 30 %. This reduction in blood glucose level is slightly more than that observed for other vanadium peroxo complexes containing ligands having O or N donor atoms[35,36].

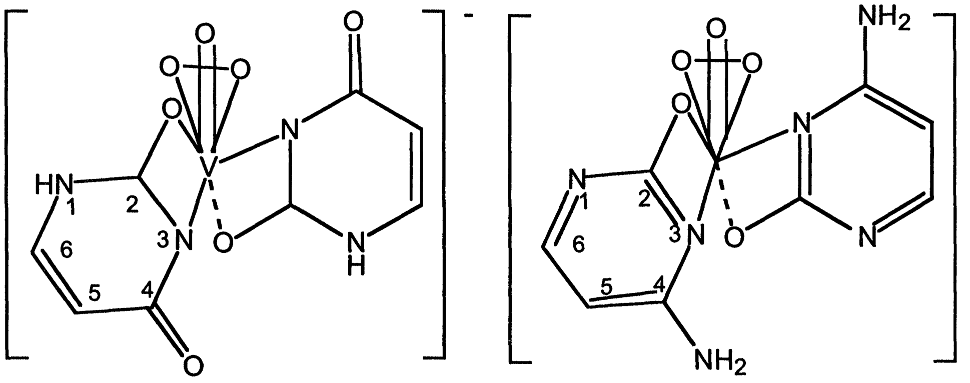

(a) (b)

Fig. 5: Proposed structure pf the uracilato (a) and cytosinato (b) complexes

Oxidation of cysteine to cystine

The reaction of the complex $K[VO(O_2)(ura)_2].H_2O$ and $K[VO(O_2)(cyt)_2].H_2O$ with cysteine solution for 4 hours under nitrogen atmosphere with molar ratios of 1:1, 2:1, and 4:1 were carried out. A fine white precipitate formed was collected and identified as cystine by its $^1H$ NMR spectrum. This conversion to cystine was presented in Table III and compared to that observed for $KVO_3$ and $H_2O_2$. The reaction with $H_2O_2$ converts almost all cysteine to cystine. This observation that the complexes (**1**) and (**2**) are much more efficient than potassium vanadate towards the oxidation of cysteine to cystine is consistent with the greater insulin mimic activity[11].

Table III: Conversion of cysteine to cystine (%)

| **Molar ratio** Cysteine : compound | Compounds (1) | (2) | $KVO_3$ | $H_2O_2$ |
|---|---|---|---|---|
| 1 : 1 | 31 | 42 | 7.9 | 95 |
| 2 : 1 | 52 | 48 | 27.5 | 95 |
| 4 : 1 | 43 | 44 | 6.8 | |

### DISCUSSION

The well characterised[35,36] insulin-mimetic oxoperoxopicolanatovanadium (V) dihydrate and the anionic pyridine-2,6-dicarboxylatooxoperoxovanadate (V) monohydrate reduces the plasma glucose level by 20 % in STZ-diabetic Sprague Dawley and insulin treated BB rats. In each case, O- or N-donor atoms were bound to two or more sites in the vanadium (V) pentagonal bipyramidal coordination sphere. The elemental analysis and conductance values supports the formulation of the isolated complexes as $K[VO(O_2)(L_2)].H_2O$ and $NH_4[VO(O_2)(L_2)].2H_2O$ where HL = uracil or cytosine. The peroxide and the uracil or cytosine ligand acts as chelates. The vanadium in the complexes will assume pentagonal bipyramidal geometry. The peroxo group, the two nitrogen and one oxygen donor atoms of both ligands forms the equatorial plane. The remaining one oxygen donor of the ligand and the oxygen of the oxo group are in the axial position (Fig. 5.)

The probable mechanism for the potent insulin mimic activity of the isolated potassium salts of the complexes may be explained as follows. Insulin interacts with the extra cellular α-subunit of the insulin receptor that is attached to the plasma membrane by the β-subunit, which passes through the membrane[37]. The peroxovanadium complexes have been shown to be effective in stimulating insulin receptor kinase (IRK) activity in hepatoma cells and inhibiting phosphotyrosine phosphatase (PTPase) activity in rat liver endosomes[3]. A very reactive cysteine residue at the active site of PTPase forms a phosphate thioester intermediate[38] during catalysis. The vanadate anion oxidises cysteine to give cystine and esterifies the phenol group of N-acetyltyrosine ethylester[39]. Hence, we have carried out the oxidation of cysteine with the isolated complexes. The observation that the complexes are more efficient oxidising agents than the potassium vanadate at the oxidative coupling of cysteine is consistent with the greater insulin mimic activity[40] of the complexes.

### ACKNOWLEDGEMENTS

The authors are grateful to Dr. P. K. Bhattacharya of the Indian Institute of Chemical Biology, Calcutta, Dr. J. N. Bera and Dr. R. K. Biswas of the Indian Institute of Science, Bangalore for recording the NMR spectra. The authors are also grateful to Dr. D. K. Mukherjee, Department of Zoology of this University for providing the laboratory facilities and fruitful discussions regarding the biochemical work. The authors express their grateful thanks to Kalyani University authorities for providing a fellowship to one of them (S.M.).

### REFERENCE

1. D. C. Crans, *in "Metal ions in Biological systems",* H. Sigel and A. Sigel, Eds., Vol. **31**, Marcel Dekker, Basel, p. 147, 1995.
2. A. Shaver, J. B. Nag, D. A. Hall, B. S. Lum and B. I. Posner, *Inorg. Chem.*, **32**, 3109, 1993.
3. B. I. Posner, R. Faure, J. W. Burgess, A. P. Bevan, D. Lachance, G. Zhang-Sun, I. G. Fantus, J. B. Ng, D. A. Hall, B. Soo Lum, A. J. Shaver, *Biol. Chem.*, **269**, 4596,1994.
4. A.Shaver, D. A. Hall, J. B. Ng, A.M. Lebius, R. C. Hynes and B. I. Posner, *Inorg. Chim. Acta,* **229**, 253, 1995.
5. H. Vilter, *in "Metal ions in Biological Systems",* H. Sigel and A. Sigel, Eds., Marcel Dekker, New York, Basel, Hong Kong, **31**, 325, 1995.
6. V. Conte, F. Di. Furia, *"Catalytic Oxidations with Hydrogen Peroxide as Oxidant"*, G. Strukul, Ed., Kluwer Academic Publishers. Dordrechet, The Netherlands, P.223, 1992.
7. V. Conte, F. Di. Furia and S. Moro, *J. Mol. Catal.,* **120**, 93, 1997.
8. Butler, M. J. Clague and G. Meister, *Chem. Rev.,* **94**, 625, 1994.
9. K. H. Thompson, J. H. McNeill and C. Orvig, *Chem. Rev.*, **99**, 2561, 1999.
10. Y. Shechter, S. J. D. Karlish, *Nature,* **284**, 556, 1980.
11. B. I. Posner, A. Shaver and I. G. Fantus, *"New Anti diabetic drugs",* C. J. Bailey and P. R. Flatt, Eds. Smith. Gordan, London, Chapter 8, p. 107, 1990.
12. A. S. Tracey, J. S. Jaswal, *J. Am. Chem. Soc.*, **114**, 3835, 1992.
13. C. Crans, A. D. Keramidas, H. Hoover-Litty, O. P. Anderson, M. M. Miller, L. M. Lemoine, S. Pleasic-Williams, M. Vandenberg, A. J. Rossomondo and L. J. Sweet, *J. Am. Chem. Soc.*, **119**, 5447, 1997.
14. Melchior, K. H. Thompson, J. M. Jong, S. J. Retting, E.Shulter, V. G. Yuen, Y. Zhou, J. H. Mc Neill and Chris Orvig, *Inorg. Chem.*, **38**, 2288, 1999.
15. C. Crans, M. Mahroof-Tahir and A. D. Keramidas, *Mol. Cell. Biochem,* **153**, 17,1995.
16. M. Halberstam, N. Cohen, P. Shlimovich, L. Rossetti and H. Shamoon, *Diabetes,* **45**, 659, 1996.
17. G. L. Eichorn, *Adv. Inorg. Biochem.*, **3**, 1, 1981.
18. L. G. Marzilli, *Adv. Inorg. Biochem.*, **3**, 47, 1981.
19. A. R. Sarkar and M. Sarkar, *J. Chem. Research (s),* 304, 1997.
20. A. R. Sarkar and M. Sarkar, *Synth. React. Inorg. Met-Org. Chem.*, **28**, 51, 1998.
21. G. Charlot and D. Bezier, *"Quantitative Inorganic Analysis"*, 3rd edn., Longman, Great Britain, p. 538, 1961.
22. H. Hyvariman and E. Nikkitia, *Clin. Chim. Acta,* **7**, 140, 1962,
23. W. P. Griffith and T. D. Wickins, *J. Chem. Soc. (A)*, 397, 1968.
24. W. Wang, F. Zeng, X. Wang and M. Tan, *Polyhedron,* **15**, 1699, 1996.
25. H. Susi and J. S. Ard, *Spectrochim Acta, A 27*, 1549, 1971.
26. H. Susi, J. S. Ard and J. M. Purcell, *Spectrochim Acta,* **29A**, 725, 1973.
27. M. Sundaralingam and J. A. Carrabine, *J. Mol. Biol.*, **61**, 287, 1971.
28. L. J. Bellary, *" The infrared spectra of complex molecule",* John Wiley, New York, 1966.

29. P. Schwendt, P. Svancarek, L. Kuchta and J. Marek, *Polyhedron,* **17,** 2161, 1998.
30. A. B. P. Lever, *J. Chem. Soc.(A),* 397, 1968.
31. K. Krogh-Jesperson, J. D. Westbrook, J. A. Potenza and J. H. Schugar, *J. Am. Chem. Soc.,* **109,** 7025, 1987.
32. R. Wagner and W. von Philipsborn, *Helv. Chim. Acta,* **53,** 299, 1970.
33. B. Lippert, *Inorg. Chem.,* **20,** 4326, 1981.
34. V. Conte, F. D. Furia and S. Moro, *Inorg. Chim. Acta,* **272,** 62, 1998.
35. J. F. Yale, D. Lachance, A. P. Bevan, C. Vigeant, A. Shaver, B. I. Posner, *Diabetes,* **44,** 1274, 1995.
36. A. P. Bevan, J. W. Burgess, J. F. Yale, P. G. Drake, D. Lachance, G. Baquiram, A. Shaver, B. I. Posner, *J. Am. Physio., E 60,* 268, 1995.
37. O. M. Rosen, *Science,* **237,** 1452, 1987.
38. D. A. Pot and J. E. Dixon, *J. Biol. Chem.,* **267,** 140, 1992.
39. A. S. Tracey and M. J. Gresser, *Proc. Natl. Acad. Sci.,* USA, **83,** 609, 1986.
40. M. J. Gresser and A. S. Tracey, *"Vanadium in biological systems"*, N. D. Chasteen, Ed. Kluwer, Dordrecht, The Netherlands, p. 63, 1990.

**Received: May 12, 2000 - Accepted: June 8, 2000 -**
**Received in publishable format: August 18, 2000**

